# Supplementary material for: Impact of Serotonin Transporter Absence on Brain Insulin Receptor Expression, Plasma Metabolome Changes, and ADHD-like Behavior in Mice fed a Western Diet
Source: Biomolecules. 2024 Jul 23;14(8):884. doi: 10.3390/biom14080884 (PMC11351952; doi:10.3390/biom14080884)
Supplement: Supplementary file 1 [file biomolecules-14-00884-s001.zip › biomolecules-3067877-supplementary.pdf]

Supplementary Material

1. Supplementary tables

Supplementary table S1. A composition of diets used.

Diet	Western Diet (WD)		Control Diet (CD)	
%	g	kcal	g	kcal
Protein	12.4	11	10.3	11
Carbohydrate	54.9	48	76.0	79
Fat	21.3	42	4.3	10
Total		100		100
kcal/gm	4.61		3.84	
**Ingredient**	**gm**	**kcal**	**gm**	**kcal**
Casein, 30 Mesh	106	424	106	424
L-Cystine	1.6	6.4	1.6	6.4
Sucrose	150	600	150	600

Maltodextrin 10	100	400	150	600
Corn Starch	216	864	481	1924
Cellulose, BW200	50	0	50	0
**Soybean Oil**	**0**	**0**	**25**	**225**
**Palm Oil**	**185**	**1665**	**20**	**180**
Mineral Mix S10026	10	0	10	0
DiCalcium Phosphate	13	0	13	0
Calcium Carbonate	5.5	0	5.5	0
Potassium Citrate, 1 H2O	16.5	0	16.5	0
Vitamin Mix V10001	10	40	10	40
Choline Bitartrate	2	0	2	0
**Cholesterol, NF**	**1.8**	**0**	**0**	**0**
**Total**	**867.45**	**3999**	**1040.65**	**3999**

Supplementary table S2. Sequences for primers used in RT-PCR.

Gene	Gene ID	Forward primer	Reverse primer
GAPDH	14433	TGCACCACCAACTGCTTAG	GGATGCAGGGATGATGTTC
ACTB	11461	GGCTGTATTCCCCTCCATCG	CCAGTTGGTAACAATGCCATGT
B2m	12010	QuantiTect Primer Assays Cat. No. QT01149547 (Qiagen, Netherlands)	
IRA	16337	GGTTTTTGTCCCCAGGCCAT	GTGCTCCTCCTGACTTGTGG
IRB	16337	CAATGGTGCCGAGGACAGTA	GTGCTCCTCCTGACTTGTGG
ACSL1	14081	ATCTGGTGGAACGAGGCAAG	TCCTTTGGGGTTGCCTGTAG
ENPP	18605	AGTGCTCCGCCTTAGAGTCC	GTCAGCAACTCCTCGACCCA
PTPN1	19246	GGCGCCAAGTTCATCATGGG	AGCTCCTTGCACTTCCCGTT
PTEN	19211	AGGCACAAGAGGCCCTAGAT	CTGACTGGGAATTGTGACTCC
CD36	12491	TCTCATGCCAGTCGGAGACA	CTGTACACAGTGGTGCCTGTT

Supplementary table S3. Bins (where VIP score > 1.5) and metabolite assignments (see the main text).

**WT/CD vs. WT/WD**		
**Bin**	**VIP score**	**Metabolite assignment**
1.32--1.34 ppm	7.638	lactate
0.86--0.88 ppm	2.892	lipid (-CH3)
4.10--4.12 ppm	2.595	lactate
3.22--3.24 ppm	2.202	β-glucose/lipid
0.84--0.86 ppm	2.131	lipid (-CH3)
1.46--1.48 ppm	2.010	alanine
3.50--3.52 ppm	1.865	glucose
3.72--3.74 ppm	1.862	glucose

**WT/CD vs. KO/CD**		
**Bin**	**VIP score**	**Metabolite assignment**
1.32--1.34 ppm	5.738	lactate
3.22--3.24 ppm	3.766	β-glucose/lipid
0.86--0.88 ppm	3.192	lipid (-CH3)
3.26--3.28 ppm	2.338	β-glucose
1.26--1.28 ppm	2.261	lipid (-CH2-)n
1.46--1.48 ppm	2.222	alanine
3.42--3.44 ppm	2.067	glucose
0.84--0.86 ppm	1.874	lipid (-CH3)

**KO/CD vs. KO/WD**		
**Bin**	**VIP score**	**Metabolite assignment**
1.32--1.34 ppm	4.958	lactate
0.86--0.88 ppm	4.026	lipid (-CH3)
0.88--0.90 ppm	3.018	lipid (-CH3)
1.28--1.30 ppm	2.817	lipid (-CH2-)n
0.84--0.86 ppm	2.811	lipid (-CH3)
3.22--3.24 ppm	2.647	β-glucose/ lipid
1.30--1.32 ppm	2.522	lipid (-CH2)n
5.29--5.31 ppm	2.521	unsaturated lipid

**WT/WD vs. KO/WD**		
**Bin**	**VIP score**	**Metabolite assignment**
0.86--0.88 ppm	4.036	lipid (-CH3)
3.22--3.24 ppm	3.126	β-glucose/ lipid
5.29--5.31 ppm	3.092	unsaturated lipid
0.88--0.90 ppm	2.933	lipid (-CH3)
1.28--1.30 ppm	2.687	lipid (-CH2-)n
3.40--3.42 ppm	2.643	glucose
1.32--1.34 ppm	2.577	lactate
5.31--5.33 ppm	2.566	unsaturated lipid

3.90--3.92 ppm	1.794	glucose
3.84--3.86 ppm	1.647	glucose
3.46--3.48 ppm	1.644	glucose
0.92--0.94 ppm	1.594	BCAA isoleucine
3.24--3.26 ppm	1.539	β-glucose/lipid

1.48--1.50 ppm	1.867	alanine
1.24--1.26 ppm	1.866	lipid (-CH2-)n
4.10--4.12 ppm	1.841	lactate
3.02--3.04 ppm	1.662	creatine/creatinine
1.04--1.06 ppm	1.521	BCAA valine

2.76--2.78 ppm	2.159	lipid (=CH-CH2-CH=)
4.10--4.12 ppm	2.098	lactate
5.31--5.33 ppm	1.967	unsaturated lipid
1.26--1.28 ppm	1.901	lipid (-CH2-)n
5.27--5.29 ppm	1.901	unsaturated lipid
2.02--2.04 ppm	1.849	lipid (=CH-CH2-CH-)
3.56--3.58 ppm	1.832	glucose
3.64--3.66 ppm	1.776	glucose
3.76--3.78 ppm	1.667	glucose
3.54--3.56 ppm	1.589	glucose
1.46--1.48 ppm	1.580	alanine

2.76--2.78 ppm	2.505	lipid (=CH-CH2-CH=)
2.02--2.04 ppm	2.229	lipid (=CH-CH2-CH-)
1.26--1.28 ppm	2.225	lipid (-CH2-)n
2.78--2.80 ppm	2.098	lipid (=CH-CH2-CH=)
0.84--0.86 ppm	2.039	lipid (-CH3)
1.30--1.32 ppm	2.001	lipid (-CH2-)n
3.26--3.28 ppm	1.871	β-glucose
5.27--5.29 ppm	1.841	unsaturated lipid
3.76--3.78 ppm	1.803	glucose
3.42--3.44 ppm	1.768	glucose
3.72--3.74 ppm	1.661	glucose

						3.78--3.80 ppm	1.576	glucose	3.46--3.48 ppm	1.607	glucose
									1.46--1.48 ppm	1.594	alanine
									1.48--1.50 ppm	1.592	alanine
									3.82--3.84 ppm	1.568	glucose

Supplementary table S4. Summary of statistical analysis of the main effects and interactions of physiological and metabolic changes, behavioral changes in the open field, brain expression of IR subtypes and transcription factors, and metabolomic parameters in wild types and Sert knockout mice fed with control diet or Western diet (see Figs. 2, 4-6, Supp. Fig. S3). One-way ANOVA. Significant group differences are in bold; ns: not significant.

Parameter	Main effects		
	Genotype effect	Diet effect	Interaction
Body weight week 1	**F=12.36, p=0.0019**	F=0.1285, p=0.7232, ns	F=0.03424, p=0.8548, ns
Body weight week 2	**F=17.29, p=0.0004**	F=2.729, p=0.1121, ns	F=0.0004869, p=0.9826, ns
Body weight week 3	**F=14.80, p=0.0009**	**F=7.261, p=0.0132**	F=0.0002291, p=0.9881, ns

Glucose tolerance AUC	F=0.7898, p=0.3882, ns	**F=10.91, p=0.0048**	F=0.1614, p=0.6935, ns
Leptin blood level	**F=10.08, p=0.0088**	**F=19.50, p=0.0010**	F=2.656, p=0.1315, ns
Mean velocity	F=3.230, p=0.0844, ns	F=0.1343, p=0.7171, ns	F=4.240, p=0.05, ns
Number of transitions	**F=7.153, p=0.0130**	F=0.574, p=0.4557, ns	F=2.06, p=0.1636, ns
Duration of stretched posture	F=2.27, p=0.1444, ns	F=1.386, p=0.2502, ns	F=1.478, p=0.2355, ns
Duration of grooming	F=3.718, p=0.0653, ns	F=0.8463, p=0.3664, ns	**F=4.517, p=0.0436**
IrA expression in hippocampus	**F=6.831, p=0.0150**	F=3.689, p=0.0662, ns	**F=12.11, p=0.0019**
IrA expression in dorsal raphe	**F=5.008, p=0.0357**	**F=7.286, p=0.0131**	**F=9.840, p=0.0048**
IrA expression in prefrontal cortex	**F=12.18, p=0.0019**	**F=5.229, p=0.0313**	F=0.9133, p=0.3488, ns
IrA expression in hypothalamus	**F=8.912, p=0.0063**	**F=4.636, p=0.0412**	**F=6.025, p=0.0214**

IrB expression in hippocampus	F=1.171, p=0.2895, ns	F=1.243, p=0.2754, ns	**F=6.729, p=0.0156**
IrB expression in dorsal raphe	**F=15.99, p=0.0007**	F=1.176, p=0.2904, ns	F=2.747, p=0.1123, ns
IrB expression in prefrontal cortex	**F=12.63, p=0.0016**	F=0.8655, p=0.3615, ns	F=1.776, p=0.1952, ns
IrB expression in hypothalamus	F=2.118, p=0.158, ns	**F=6.359, p=0.0184**	**F=15.4, p=0.0006**
ACSL1 expression in hippocampus	F=2.894, p=0.1052, ns	F=0.4987, p=0.4887, ns	**F=7.709, p=0.012**
ACSL1 expression in dorsal raphe	F=3.839, p=0.0649, ns	**F=4.978, p=0.0379**	F=0.5091, p=0.4842, ns
ACSL1 expression in prefrontal cortex	F=2.88, p=0.106, ns	F=0.09974, p=0.758, ns	F=0.3635, p=0.5537, ns
ACSL1 expression in hypothalamus	F=0.1008, p=0.7543, ns	F=0.698, p=0.4138, ns	F=0.3041, p=0.5878, ns
ENPP expression in hippocampus	**F=13.42, p=0.0017**	F=2.153, p=0.1587, ns	F=4.066, p=0.0581, ns
ENPP expression in	F=0.8958, p=0.3558, ns	F=2.338, p=0.1428, ns	F=3.936, p=0.0619, ns

dorsal raphe			
ENPP expression in prefrontal cortex	F=0.7062, p=0.4111, ns	F=0.1466, p=0.7061, ns	F=0.8035, p=0.3813, ns
ENPP expression in hypothalamus	F=0.2164, p=0.6471, ns	F=0.019, p=0.8918, ns	F=0.05743, p=0.8132, ns
PTPN1 expression in hippocampus	F=3.308, p=0.0847, ns	F=1.993, p=0.1742, ns	**F=11.88, p=0.0027**
PTPN1 expression in dorsal raphe	**F=9.489, p=0.0062**	F=3.044, p=0.0972, ns	F=0.002973, p=0.9571, ns
PTPN1 expression in prefrontal cortex	F=0.1376, p=0.7147, ns	F=3.059, p=0.0964, ns	F=0.01529, p=0.9029, ns
PTPN1 expression in hypothalamus	F=0.1825, p=0.6740, ns	**F=5.837, p=0.0259**	F=0.1285, p=0.7239, ns
PTEN expression in hippocampus	F=1.762, p=0.2001, ns	**F=8.608, p=0.0085**	F=0.2820, p=0.6016, ns
PTEN expression in dorsal raphe	**F=4.693, p=0.0432**	F=0.5955, p=0.4498, ns	F=0.4606, p=0.5055, ns
PTEN expression in prefrontal cortex	**F=7.579, p=0.0127**	F=4.377, p=0.0501, ns	F=0.1866, p=0.6707, ns

PTEN expression in hypothalamus	F=0.9680, p=0.3375, ns	F=0.03909, p=0.8454, ns	F=0.002877, p=0.9578, ns
CD36 expression in hippocampus	F=0.8532, p=0.3672, ns	F=1.194, p=0.2881, ns	F=0.002275, p=0.9625, ns
CD36 expression in dorsal raphe	F=0.2069, p=0.6543, ns	F=0.1694, p=0.6852, ns	F=0.006875, p=0.9348, ns
CD36 expression in prefrontal cortex	F=1.202, p=0.2867, ns	F=0.1550, p=0.6981, ns	F=0.5186, p=0.4802, ns
CD36 expression in hypothalamus	F=2.994, p=0.0998, ns	F=0.7086, p=0.4104, ns	F=2.234, p=0.1514, ns
Lipid	**F=13.61, p=0.0014**	F=0.6235, p=0.4386, ns	F=3.071, p=0.0943, ns
Unsaturated lipid	F=3.014, p=0.0965, ns	F=2.758, p=0.1109, ns	**F=4.339, p=0.0491**
HDL	F=0.135, p=0.7168, ns	F=3.249, p=0.0852, ns	F=1.248, p=0.276, ns
VLDL	**F=4.906, p=0.0379**	**F=8.572, p=0.008**	F=0.7989, p=0.3815, ns
Glucose	**F=16.38, p=0.0006**	F=0.02511, p=0.8756, ns	**F=9.263, p=0.0062**
Lactate	F=3.433, p=0.0774, ns	**F=16.05, p=0.0006**	F=0.04025, p=0.8428, ns

Alanine	**F=25.95, p<0.0001**	**F=24.63, p<0.0001**	F=0.02009, p=0.8886, ns
Isoleucine	**F=8.356, p=0.0085**	**F=27.56, p<0.0001**	F=0.5327, p=0.4732, ns
Valine	**F=20.69, p=0.0002**	**F=17.96, p=0.0004**	F=0.1263, p=0.7258, ns

Supplementary table S5. Summary of statistical values of multiple comparisons for parameters with significant genotype x diet interaction: behavioral changes in the open field, brain expression of IR subtypes and transcription factors, and metabolomic parameters in wild types and Sert knockout mice fed with control diet or Western diet (Figs. 4-6, Supp. Fig. S3). *Post-hoc* Tukey's test. Significant differences are in bold; ns: not significant. WT - wild type, KO - knockouts, CD - control diet, WD - Western diet.

Parameter	post hoc Tukey's test			
	WT/CD vs. WT/WD	WT/CD vs. KO/CD	KO/CD vs. KO/WD	WT/WD vs. KO/WD
Duration of grooming	p=0.1982, ns	**p=0.0442**	p=0.804, ns	p=0.9989, ns
IrA expression in hippocampus	p=0.7231, ns	**p=0.0015**	**p=0.0024**	p=0.9277, ns

IrA expression in dorsal raphe	p=0.9914, ns	**p=0.0071**	**p=0.001**	p=0.9098, ns
IrA expression in hypothalamus	p=0.997, ns	**p=0.0047**	**p=0.0102**	p=0.9804, ns
IrB expression in hippocampus	p=0.754, ns	p=0.0769, ns	**p=0.0475**	p=0.6972, ns
IrB expression in hypothalamus	p=0.7824, ns	**p=0.0052**	**p=0.0003**	p=0.3044, ns
ACSL1 expression in hippocampus	p=0.1098, ns	p=0.8785, ns	p=0.4569, ns	**p=0.0294**
PTPN1 expression in hippocampus	**p=0.016**	p=0.6791, ns	p=0.4711, ns	**p=0.0059**
Unsaturated lipids	p=0.9905, ns	p=0.9952, ns	p=0.0653, ns	**p=0.0466**
Glucose	p=0.1875, ns	p=0.9074, ns	p=0.1535, ns	**p=0.0001**

Supplementary table S6. Summary of t-test and one-sample t-test statistical values for of physiological and metabolic changes, behavioral changes in the open field, brain expression of IR subtypes and transcription factors, and metabolomic parameters in wild types and Sert knockout mice fed with control diet or Western diet (Figs. 2, 4-6, Supp. Fig. S3). Significant differences are in bold; ns: not significant, WT - wild type, KO – knockouts.

Parameter	Paired comparison	Comparison with 100%	
		WT	KO
Body weight week 1	p=0.7617, ns	p=0.6068, ns	p=0.8536, ns
Body weight week 2	p=0.8218, ns	p=0.1812, ns	p=0.0833, ns
Body weight week 3	p=0.7939, ns	p=0.0745, ns	**p=0.0075**
Glucose tolerance AUC	p=0.8193, ns	**p=0.0279**	p=0.0569, ns
Leptin blood level	**p=0.0013**	**p=0.0449**	**p=0.0087**
Mean velocity	**p=0.0038**	**p=0.0359**	**p=0.0264**
Number of	p=0.1034, ns	p=0.4470, ns	p=0.1336, ns

transitions			
Duration of stretched posture	p=0.1846, ns	**p<0.0001**	p=0.2202, ns
Duration of grooming	**p=0.0034**	**p=0.0213**	p=0.1148, ns
IrA expression in hippocampus	**p=0.0006**	p=0.1460, ns	**p=0.0004**
IrA expression in dorsal raphe	**p=0.0098**	p=0.7961, ns	**p<0.0001**
IrA expression in prefrontal cortex	p=0.6401, ns	**p=0.0030**	**p=0.0334**
IrA expression in hypothalamus	**p=0.0041**	p=0.6540, ns	**p=0.0021**
IrB expression in hippocampus	**p=0.0022**	p=0.2004, ns	**p=0.0005**
IrB expression in dorsal raphe	**p=0.0446**	p=0.4758, ns	**p=0.0429**
IrB expression in prefrontal cortex	p=0.0605, ns	p=0.6350, ns	**p=0.0497**

IrB expression in hypothalamus	**p=0.0012**	p=0.3514, ns	**p<0.0001**
ACSL1 expression in the hippocampus	**p=0.0031**	**p=0.0293**	p=0.0578, ns
ACSL1 expression in dorsal raphe	p=0.1602, ns	p=0.1027, ns	**p=0.0278**
ACSL1 expression in prefrontal cortex	p=0.3577, ns	p=0.8114, ns	p=0.1973, ns
ACSL1 expression in hypothalamus	p=0.4692, ns	p=0.4075, ns	p=0.3804, ns
ENPP expression in hippocampus	p=0.1650, ns	**p=0.0064**	p=0.7674, ns
ENPP expression in dorsal raphe	**p=0.0190**	p=0.6250, ns	**p=0.0345**
ENPP expression in prefrontal cortex	p=0.2582, ns	p=0.7106, ns	p=0.1322, ns
ENPP expression in hypothalamus	p=0.7256, ns	p=0.4389, ns	p=0.9393, ns

PTPN1 expression in hippocampus	**p=0.0006**	**p=0.0074**	p=0.0971, ns
PTPN1 expression in dorsal raphe	p=0.8898, ns	p=0.1054, ns	p=0.2781, ns
PTPN1 expression in prefrontal cortex	p=0.8972, ns	p=0.1914, ns	p=0.1465, ns
PTPN1 expression in hypothalamus	p=0.6591, ns	**p=0.0481**	p=0.0759, ns
PTEN expression in hippocampus	p=0.7976, ns	**p=0.0190**	p=0.0581, ns
PTEN expression in dorsal raphe	p=0.4624, ns	p=0.8873, ns	p=0.3937, ns
PTEN expression in prefrontal cortex	p=0.9828, ns	**p=0.0111**	**p=0.0236**
PTEN expression in hypothalamus	p=0.9641, ns	p=0.6591, ns	p=0.8567, ns
CD36 expression in hippocampus	p=0.2858, ns	**p=0.0293**	p=0.1889
CD36 expression in	p=0.9414, ns	p=0.7535, ns	p=0.6780, ns

dorsal raphe			
CD36 expression in prefrontal cortex	p=0.2348, ns	p=0.7491, ns	p=0.1657, ns
CD36 expression in hypothalamus	p=0.1722, ns	p=0.0690, ns	p=0.6611, ns

2. Supplementary figures

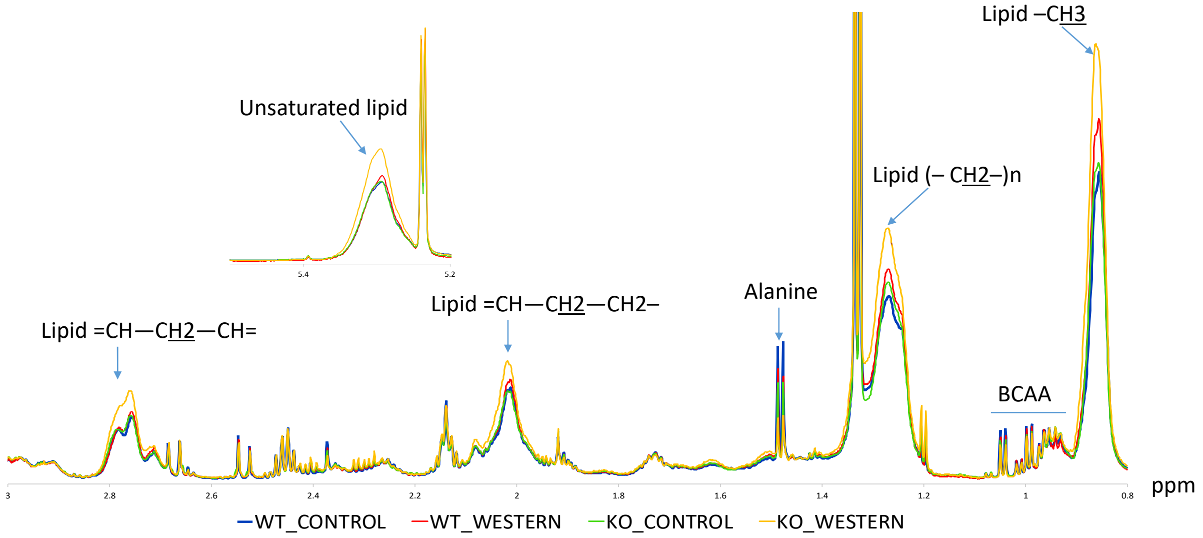

Supplementary Figure S1. Average ^{1}H CPMG spectra of WT/CD (blue), WT/WD (red), KO/CD (green), and KO/WD (yellow). Lipid signals as well as BCAAs and alanine are shown. Hydrogens that are responsible for the NMR signal are underlined.

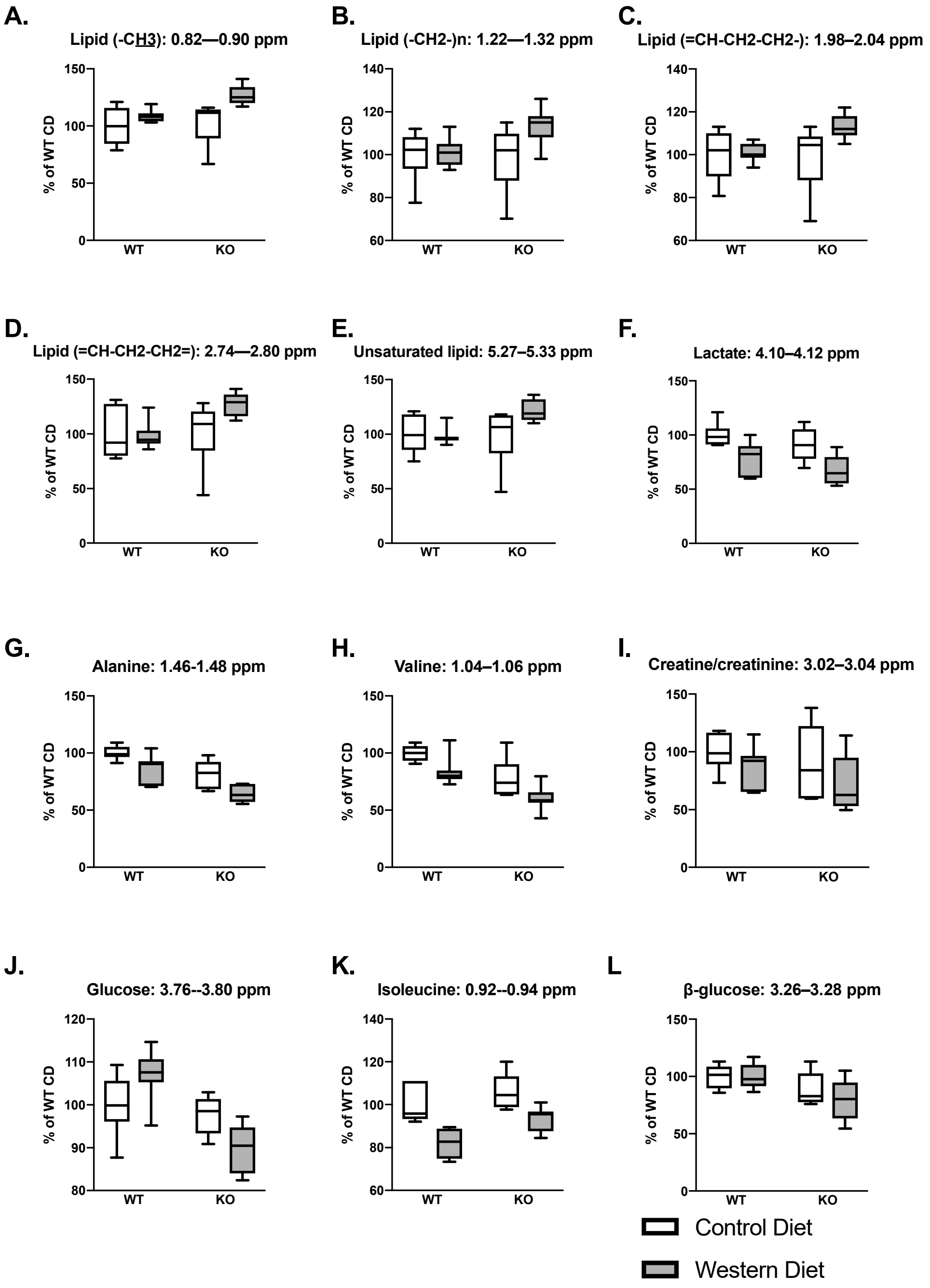

Supplementary Figure S2. Boxplots showing average percent change of each metabolite signal relative to the WT/CD group are shown. **(A)** Average percent change of lipid (-CH3) was higher in WD-fed groups compared to the WT/CD group. Average percent change of **(B)** lipid (-CH2), **(C)** lipid (=CH-CH2-CH2-), **(D)** lipid (=CH-CH2-CH2=), and **(E)** unsaturated lipid was higher in KO/WD group compared to the WT/CD group. **(F)**

Average percent change of lactate was lower in WD-fed groups compared to the WT/CD group. Average percent change of **(G)** alanine, **(H)** valine, and **(I)** creatine/creatinine was lower in both WD-fed groups and KO/CD group compared to the WT/CD group. **(J)** Average percent change of glucose was higher in WT/WD group, but lower in KO/WD group compared to the WT/CD group. **(K)** Average percent change of isoleucine was lower in WD-fed groups compared to the WT/CD group. **(L)** Average percent change of β-glucose was lower in KO groups compared to the WT/CD group. The regions of the spectrum used to calculate the integrals are indicated in ppm.

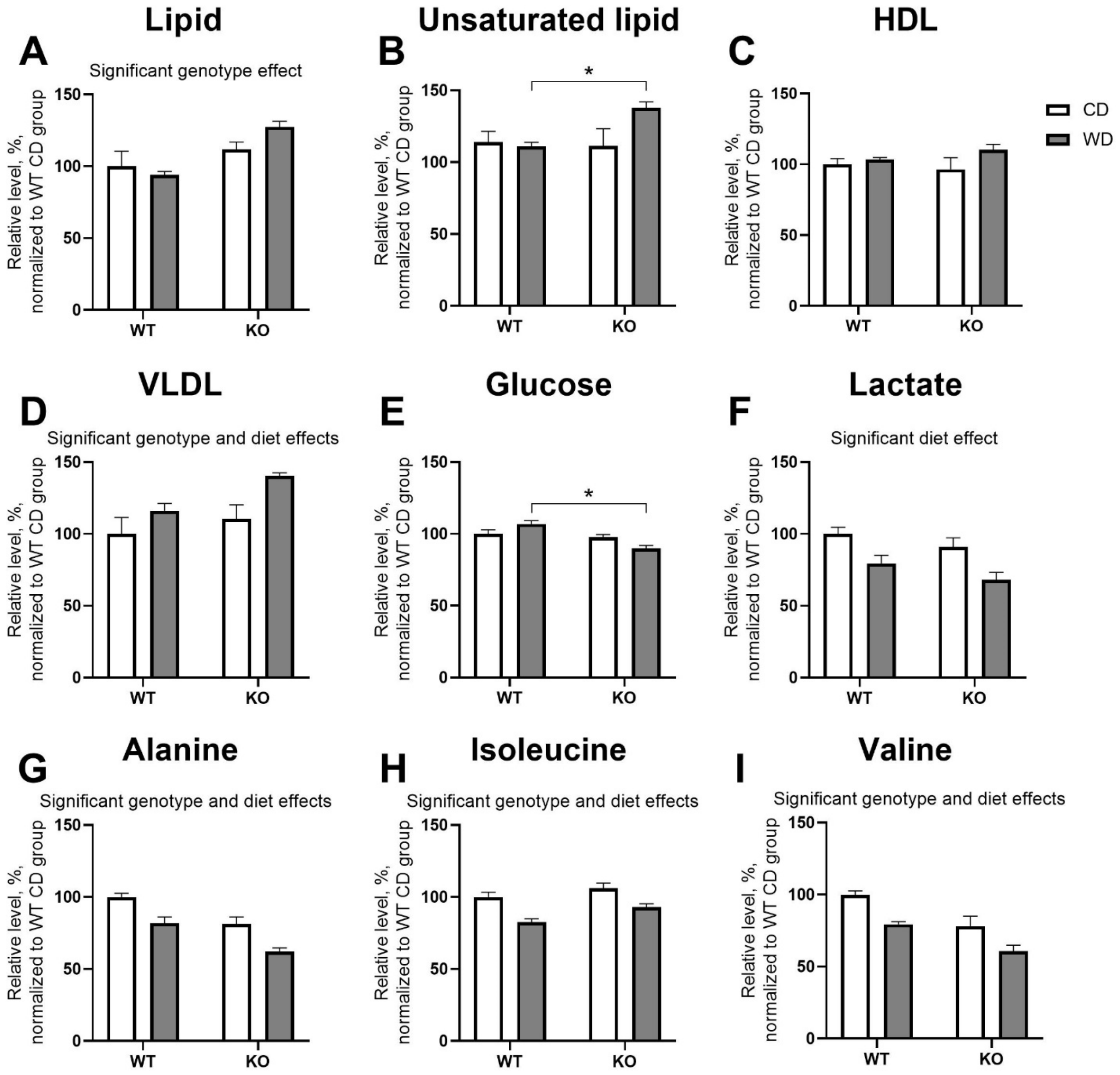

Supplementary Figure S3. Metabolome changes between SERT-KO and WT mice housed on WD. (A) Significant genotype effect was found in lipid level. **(B)** Unsaturated lipid level was significantly higher in KO/WD mice compared to WT/WD group. **(C)** No significant group differences were found in HDL level. **(D)** Significant genotype and diet effects were shown for VLDL level. **(E)** Glucose concentration was significantly lower in KO/WD mice compared to WT/WD group. **(F)** Significant diet effect was revealed in lactate level. **(G)** There was a significant genotype and diet effects on alanine level, as well as on **(H)** isoleucine content and **(I)** valine concentration. Two-way ANOVA and Tukey's post hoc test (*$p<0.05$). All data are mean±SEM.
